# miR-452-3p Targets Adenomatous Polyposis Coli to Regulate the Malignant Phenotypes and Prognosis of Esophageal Cancer

**DOI:** 10.5152/tjg.2026.26023

**Published:** 2026-05-13

**Authors:** Dian Lin, Yaqiong Xie, Yaxi Song, Miao He, Xingwu Huang

**Affiliations:** 1 Chongqing Jialing Hospital, Chongqing, China; 2 Department of Oncology, Jianli People’s Hospital, Jingzhou, China; 3 The First Department of Oncology, Xinxiang Central Hospital, The Fourth Clinical College of Xinxiang Medical University, Xinxiang, China; 4 Clinical Diagnosis and Treatment Center for Digestive Diseases (Gastrointestinal Oncology Group), The Affiliated People's Hospital of Chongqing Three Gorges Medical College, Chongqing, China; 5 Department of Oncology, Xiangyang Hospital of Traditional Chinese Medicine, Xiangyang, China

**Keywords:** Adenomatous polyposis coli, esophageal cancer, miR-452-3p, prognosis

## Abstract

**Background/Aims:**

: Esophageal cancer is a common malignancy of the digestive tract worldwide, with extremely poor prognosis in advanced stages. miR-452-3p regulates multiple tumors and may contribute to the development and progression of esophageal cancer. The current study aims to examine the correlation between miR-452-3p expression and prognosis in esophageal cancer and further investigate the potential molecular mechanisms by which miR-452-3p regulates the malignant phenotype of esophageal cancer cells.

**Materials and Methods:**

: A total of 155 pairs of esophageal cancer tissues and adjacent normal tissue samples were collected. Reverse Transcription Quantitative PCR was used to detect the expression levels of miR-452-3p and adenomatous polyposis coli (APC). Survival analyses were performed using Kaplan–Meier and Cox regression analyses. The effects of miR-452-3p on the biological behavior of esophageal cancer cells were assessed through in vitro experiments. Additionally, dual-luciferase reporter gene assays and rescue experiments were performed to confirm the regulatory relationship between miR-452-3p and APC.

**Results:**

The miR-452-3p expression is upregulated in esophageal cancer. Elevated miR-452-3p expression is closely related to the clinical pathological characteristics and poor prognosis of esophageal cancer and serves as an independent prognostic risk factor. Functional experiments demonstrate that miR-452-3p overexpression enhances the malignant biological behavior of esophageal cancer cells. miR-452-3p directly targets APC. Silencing APC reverses the inhibitory effect of miR-452-3p inhibitor on the malignant phenotype.

**Conclusion:**

miR-452-3p promotes esophageal cancer progression by targeting APC, and patients with low levels of miR-452-3p exhibit favorable prognosis.

**Main Points**

- miR-452-3p is highly expressed in esophageal cancer tissues and cells.
- miR-452-3p is associated with the malignant progression and poor prognosis of esophageal cancer.
- miR-452-3p promotes the malignant biological behaviors of esophageal cancer cells.
- miR-452-3p directly targets adenomatous polyposis coli (APC) and inhibits its expression, and the 2 are negatively correlated in cancer tissues.
- Silencing APC can reverse the effect of miR-452-3p inhibition on the malignant phenotype of cancer cells.

## Introduction

Esophageal cancer is a highly invasive malignancy of the gastrointestinal tract, which is characterized by late diagnosis, rapid progression, and high mortality.^1^^,^^2^ Its strong invasiveness and lethality impose a substantial and persistent global health burden.^3^ Epidemiological studies have shown significant geographical variations in the incidence of esophageal cancer worldwide.^4^ According to GLOBOCAN 2020 statistics, the global annual incidence of esophageal cancer exceeds 600 000 cases, with the highest incidence rates concentrated in East Asia.^5^ Notably, China bears more than 50% of the global disease burden, making it a key region for esophageal cancer prevention and treatment.^6^ Despite advances in surgical resection, chemotherapy, and radiotherapy, patient survival remains suboptimal, primarily because of delayed diagnosis and rapid disease progression.^7^^,^^8^ Therefore, identifying novel molecular targets is crucial for improving the clinical efficacy of esophageal cancer treatment.

MicroRNAs (miRNAs) participate in various biological processes and are closely associated with tumor progression.^9^ Accumulating evidence suggests that dysregulated miRNAs significantly influence the progression of esophageal cancer.^10^^,^^11^ For instance, miR-21 is overexpressed in esophageal cancer and promotes tumor growth by targeting TPM1,^12^ whereas miR-375 acts as a tumor suppressor by inhibiting PRDX1.^13^ Additionally, miR-21 and miR-375 are closely associated with the prognosis of esophageal cancer.^14^^,^^15^ The lncRNA ST7-AS1/miR-4262 axis represents a potential therapeutic biomarker for patients with esophageal cancer.^16^ Notably, miR-452-3p has been shown to be upregulated in hepatocellular carcinoma and is correlated with adverse prognostic outcomes by targeting CPEB3.^17^ It is also involved in the development of cervical cancer.^18^ Levels of miR-452-3p are closely associated with the epithelial–mesenchymal transition process in colorectal cancer.^19^ Importantly, miR-452-3p is aberrantly expressed in esophageal cancers, suggesting its potential as a novel miRNA biomarker.^20^ However, its expression profile, clinical significance, and functional role in esophageal cancer remain largely unexplored.

The current research intends to examine the correlation between miR-452-3p levels in esophageal cancer tissues and clinicopathological features as well as patient prognosis and to further investigate its potential mechanism of action in esophageal cancer progression.

## Materials and Methods

### Clinical Samples

A total of 155 cases of surgically resected tissue specimens (cancer tissues) from patients with pathologically confirmed esophageal cancer and corresponding adjacent normal tissues (at least 5 cm away from the tumor focus, pathologically confirmed to be free of cancer cells) were collected from Xinxiang Central Hospital Hospital from September 2017 to April 2020. All patients were pathologically confirmed to have esophageal cancer, and their tissue specimens were preserved following standard protocols, ensuring they were free from contamination. None of the patients had undergone radiotherapy or chemotherapy before surgery, and all had complete clinical data. During the 60-month postoperative follow-up period conducted via online access or outpatient visits, patients’ survival status was comprehensively and meticulously documented. Death was defined as the endpoint event. The exclusion criteria included patients with impaired function of vital organs, including the heart, liver, and kidneys, concurrent autoimmune diseases or mental health disorders, other malignant tumors, and prior cancer-related treatments.

This study was approved by the Xinxiang Central Hospital Hospital (approval number: 2017-012, July 14, 2017) Ethics Committee, and all patients signed informed consent forms.

### RT-qPCR

Total RNA was extracted using TRIzol reagent (Invitrogen, Carlsbad, California, USA). RNA was reverse-transcribed into cDNA using a PrimeScript RT Reagent Kit (TaKaRa, Kusatsu, Shiga, Japan). RT-qPCR was performed using a SYBR Premix Ex Taq Kit (TaKaRa) on a StepOnePlus Real-Time PCR System (Thermo Fisher Scientific, Carlsbad, California, USA). U6 served as the internal reference for miR-452-3p and GAPDH for adenomatous polyposis coli (APC) mRNA.

### Cell Culture and Transfection

Human normal esophageal epithelial cells (HEECs) and human esophageal cancer cell lines (OE33, OE19, KYSE150, and TE-1) were purchased from the Shanghai Cell Research Center, Chinese Academy of Sciences. All cells were cultured in Roswell Park Memorial Institute (RPMI) 1640 medium (Gibco, Waltham, Massachusetts, USA) supplemented with 10% Fetal Bovine Serum(FBS) at 37°C with 5% CO_2_.

miR-452-3p mimic, miR-452-3p inhibitor, negative control (NC) mimic, NC inhibitor, and si-APC were synthesized by GenePharma (Shanghai, China). Cell transfection was performed using Lipofectamine 3000 reagent, following the manufacturer’s instructions. Transfection efficiency was detected 48 hours after transfection to ensure the reliability of subsequent experiments.

### Cell Counting Kit‑8 Assay

Cell proliferation ability was detected using the CCK-8 assay (CA1210, Solarbio, China). Transfected cells were seeded at a density of 5 × 10³ cells/well into a 96-well plate. CCK-8 reagent was introduced to each well. After further incubation for 4 hours, the absorbance was measured at a wavelength of 450 nm.

### Transwell Assay

For the migration assay, 5 × 10⁴ cells/mL in 100 µL of serum-free RPMI 1640 were seeded into Transwell upper chambers , with 500 µL of serum-supplemented medium =in the lower chambers. After 24 hours, non-migrated cells were removed and migrated cells were fixed, stained with crystal violet, and counted under a microscope. For the invasion assay, Matrigel (1 : 8 dilution in RPMI 1640, 50 µL) was coated onto upper chambers and solidified at 37°C for 5 hours, with subsequent steps performed as per the migration assay.

### Dual-Luciferase Reporter Gene Assay

TargetScan was used to predict the potential binding site between miR-452-3p and APC. Luciferase expression vectors of APC-WT and APC-MUT were constructed (Genomeditech, Shanghai, China). Cells were cultured in 24-well plates at a density of 5 × 10⁴ cells/well followed by the transfection of the aforementioned reporter vectors using Lipofectamine 2000 reagent. After the 48-h post-transfection incubation, relative luciferase activity was measured using a dual-luciferase reporter assay system.

### RNA Immunoprecipitation

RNA immunoprecipitation (RIP) experiments were performed using the Magna RIP RNA-Binding Protein Immunoprecipitation Kit (Millipore, Burlington, Massachusetts, USA). Lysates from KYSE150 and TE-1 cells were used to release RNA–protein complexes. Immunoprecipitation was performed using RBP-specific antibodies and protein A/G magnetic beads, followed by thorough washing to purify co-precipitated RNA. The purified precipitated RNA was analyzed through RT-qPCR to detect the abundance of miR-452-3p and APC.

### Statistical Analysis

Statistical Product and Service Solutions (SPSS) 26.0 (IBM SPSS Corp.; Armonk, NY, USA) was used for statistical analyses. Measurement data (mean ± SD) were compared by the *t*-test and enumeration data by the chi-square test.

## Results

### Expression of miR-452-3p in Esophageal Cancer and Its Clinical Significance

**Figure 1:**
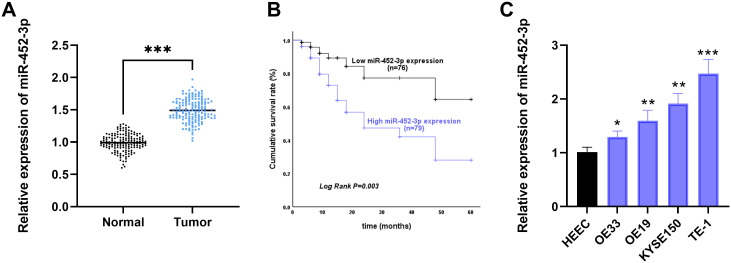
Expression of miR-452-3p in esophageal cancer and its relationship with patient prognosis. (A) Relative expression of miR-452-3p in esophageal cancer tissues and adjacent normal tissues. (B) Kaplan–Meier survival curves of patients with high vs. low miR-452-3p expression. (C) The miR-452-3p expression in esophageal cancer cell lines and HEEC. *P < .05, **P < .01, ***P < .001. HEEC, human normal esophageal epithelial cell.

RT-qPCR demonstrated markedly elevated relative miR-452-3p levels in esophageal cancer tissues (Figure 1A). Patients were stratified into high-expression (n = 79) and low-expression (n = 76) subgroups using the median miR-452-3p level in tumor tissues as the cutoff value. Correlation analysis revealed that elevated miR-452-3p expression was significantly associated with advanced tumor–node–metastasis staging (*P *= .026) and lymph node metastasis (*P *= .019). However, no statistically significant associations were observed between miR-452-3p expression and patient gender, age, tumor size, or tumor differentiation (*P *> .05) (Table 1).

**Figure 2:**
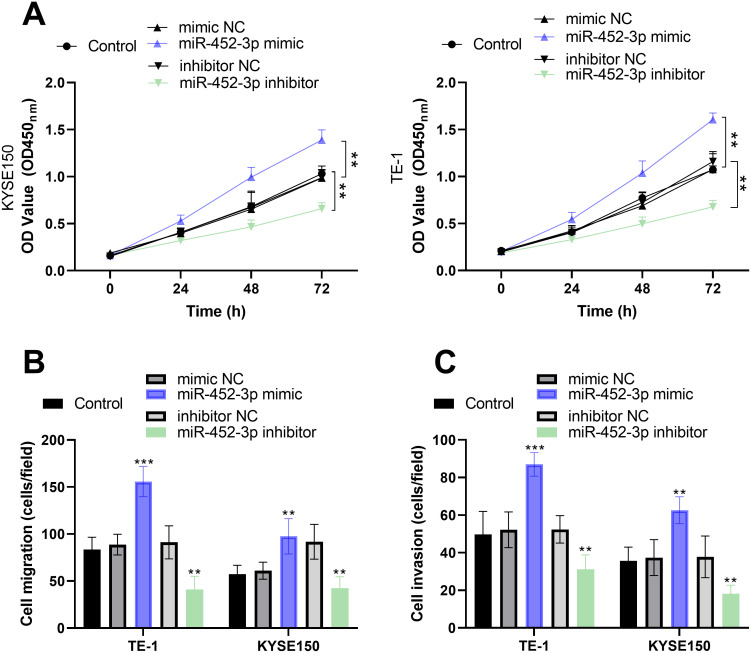
Effect of miR-452-3p on biological behaviors of esophageal cancer cells. (A–C) Effects of miR-452-3p on cell proliferation, migration, and invasion. (n = 3). **P < .01, ***P < .001.

The Kaplan–Meier survival curve indicates that low miR-452-3p expression is associated with a higher survival rate (log-rank *P *= .003, Figure 1B). Furthermore, multivariate Cox regression analysis demonstrated that high miR-452-3p expression is an independent risk factor for poor prognosis in patients with esophageal cancer (HR = 2.653, 95% CI = 1.189-5.916, *P *= .017, Table 2).Figure 2

### Effect of miR-452-3p on Esophageal Cancer Cells

RT-qPCR results showed that the miR-452-3p expression in esophageal cancer cell lines was significantly higher than that in HEEC, with the most significant upregulation observed in KYSE150 and TE-1 cells (Figure 1C). Consequently, these cells were chosen for subsequent functional experiments. Transfection with miR-452-3p mimic significantly increased the miR-452-3p expression, whereas transfection with miR-452-3p inhibitor decreased its expression (Supplementary Figure 1A). Compared with control, the proliferation ability of cells in the miR-452-3p mimic group was substantially enhanced, and the migration and invasion abilities were also substantially improved; conversely, the proliferation, migration, and invasion abilities of cells in the miR-452-3p inhibitor group were substantially weakened (Figures 2A-C).

### Molecular Mechanism of miR-452-3p in Targeting and Regulating Adenomatous Polyposis Coli

**Figure 3:**
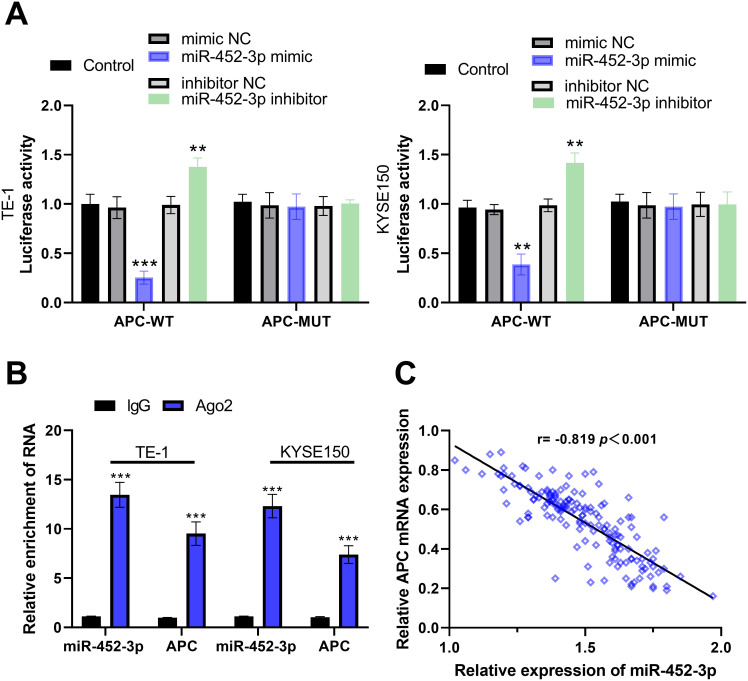
Verification of the targeted regulatory relationship between miR-452-3p and APC. (A) Dual-luciferase reporter gene assay results. (B) RIP assay results. (C) Correlation between miR-452-3p and APC mRNA expression. (n = 3). **P < .01, ***P < .001. APC, adenomatous polyposis coli; RIP, RNA immunoprecipitation.

TargetScan prediction revealed a potential binding site between APC and miR-452-3p (Supplement Figure 2A). When miR-452-3p mimic was co-transfected with APC-WT, luciferase activity was significantly reduced. In contrast, co-transfection with MUT-APC resulted in no significant change in luciferase activity, indicating that miR-452-3p directly targets APC (Figure 3A). The enrichment of miR-452-3p and APC in the Ago2 protein was detected using RIP assays. Compared to the IgG control group, the relative RNA enrichment levels of miR-452-3p and APC were significantly elevated in the Ago2 group, further confirming their targeted binding relationship (Figure 3B). APC levels in esophageal cancer tissues were significantly lower than those in adjacent normal tissues (Supplementary Figure 2B). Moreover, a significant negative correlation was observed between APC and miR-452-3p expression (*r* = −0.819, *P *< .001, Figure 3C).

### miR-452-3p Regulates Malignant Phenotypes of Esophageal Cancer Cells by Targeting Adenomatous Polyposis Coli

**Figure 4:**
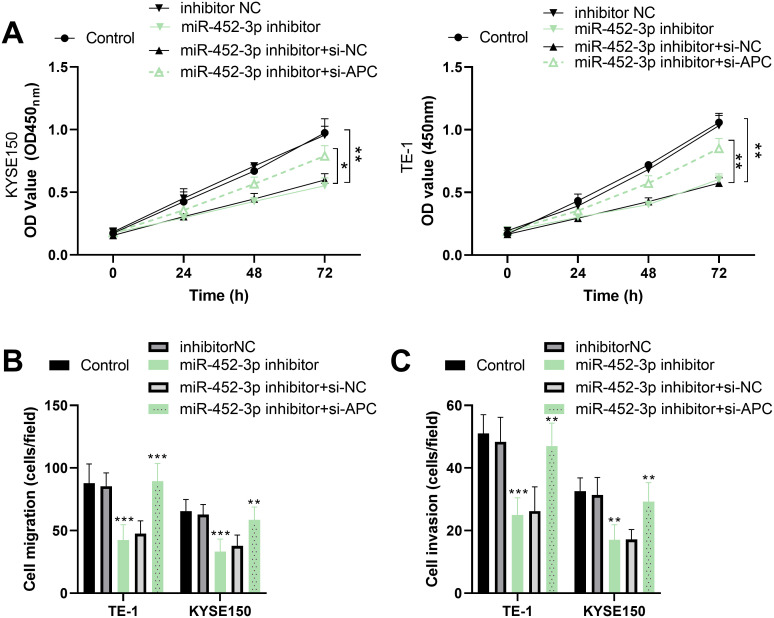
Effect of miR-452-3p/APC on biological behaviors of esophageal cancer cells. (A–C) Effects of miR-452-3p inhibitor combined with si-APC on cell proliferation, migration, and invasion (n = 3). *P < .05, **P < .01, ***P < .001. APC, adenomatous polyposis coli.

Compared to the control group, transfection with miR-452-3p inhibitor significantly upregulated APC mRNA levels. This upregulation was effectively reversed upon co-transfection with si-APC, resulting in a significant reduction in APC mRNA levels (Supplementary Figure 1B). Functional assays further corroborated that the proliferation, migration, and invasion capabilities of cells in the miR-452-3p inhibitor group were substantially diminished. However, co-transfection with the miR-452-3p inhibitor and si-APC markedly restored cell proliferation ability, accompanied by a significant increase in the number of transmembrane cells during migration and invasion (Figure 4A-C).

## Discussion

Esophageal cancer ranks among the most aggressive malignancies worldwide.^21^ The identification of novel biomarkers, coupled with a comprehensive understanding of its molecular mechanisms, holds great promise for improving patient survival rates.^22^ We systematically elucidated the functional role of miR-452-3p in esophageal cancer and identified a novel miR-452-3p/APC regulatory axis that promotes malignant progression and is associated with poor clinical prognosis in patients with esophageal cancer. Collectively, the study’s findings provide critical mechanistic insights into the molecular pathogenesis of esophageal cancer.

miRNAs have been widely recognized as key regulators of tumor progression, and their abnormal expression is closely associated with the malignant phenotypes of cancer cells.^23^^,^^24^ For example, miR-675-3p promotes esophageal cancer progression by regulating downstream signaling pathways.^25^ miR-495-3p promotes carcinogenesis by regulating the proliferation and migration of esophageal cancer cells.^26^ The results demonstrated that miR-452-3p is significantly upregulated in esophageal cancer. Its high expression correlates with aggressive clinicopathological features, suggesting a pro-oncogenic role in esophageal cancer progression. Integrative Kaplan–Meier and Cox regression analyses identified miR-452-3p as an independent prognostic factor, underscoring its potential as a precision biomarker for risk stratification and outcome prediction in esophageal cancer. This finding is consistent with that of previous studies showing that miR-452-3p is upregulated in hepatocellular carcinoma and that patients with low levels of miR-452-3p exhibit improved prognostic survival.^27^ Functional experiments further confirmed the oncogenic role of miR-452-3p in esophageal cancer. Overexpression of miR-452-3p enhances the proliferation, migration, and invasion abilities of esophageal cancer cells, whereas inhibition of miR-452-3p exerts the opposite effects. Collectively, the data demonstrated that miR-452-3p promotes tumor progression by enhancing key hallmarks of cancer, including dysregulated proliferation and metastatic dissemination.

Identifying the downstream target genes of miRNAs is crucial for clarifying their regulatory mechanisms in tumors.^28^ miR-126 is downregulated in esophageal cancer and functions as a tumor suppressor by targeting Vascular Endothelial Growth Factor‑A (VEGF-A).^29^ In this study, the authors confirmed that APC is a target of miR-452-3p. APC is a tumor-suppressor gene that plays a key role in regulating the Wnt/β-catenin signaling pathway.^30^^,^^31^ In colorectal cancer, APC mutations are an early event in the adenoma–carcinoma transformation, with loss of function promoting tumor initiation and progression through activation of the Wnt signaling pathway.^32^^,^^33^ Additionally, hypermethylation of the APC promoter has been detected in both esophageal adenocarcinoma and squamous cell carcinoma tissues.^34^ Reduced APC level increases β-catenin and β-catenin-mediated expression of cyclin D1 and PKM2, thereby promoting malignant behavior in esophageal cancer cells.^35^ Analysis of clinical samples demonstrated that APC is significantly downregulated in esophageal cancer tissues and negatively correlates with miR-452-3p expression. Rescue experiments further confirmed that APC silencing abrogated the suppressive effects of miR-452-3p knockdown on malignant phenotypes of esophageal cancer cells. miR-452-3p may induce low expression or functional loss of APC by direct targeting, thereby activating the Wnt/β-catenin signaling pathway and promoting esophageal cancer progression. However, the specific regulatory mechanism remains to be investigated.

This regulatory relationship is further supported by the inverse correlation between miR-452-3p and APC expression in clinical samples and functional rescue experiments. Specifically, silencing APC reverses the inhibitory effects of miR-452-3p depletion on esophageal cancer cell malignancy. It is thus hypothesized that in esophageal cancer, miR-452-3p directly targets APC to induce its downregulation or functional inactivation, which in turn causes nuclear accumulation of β-catenin and activation of downstream target genes, ultimately promoting cell proliferation, migration, and invasion.

The clinical implications of the findings focus on prognosis and therapy. As an independent prognostic factor, miR-452-3p could complement existing clinicopathological indicators to improve risk stratification in patients with esophageal cancer. For example, elevated miR-452-3p may identify patients at high risk of recurrence, warranting more aggressive adjuvant therapies. Therapeutically, miR-452-3p represents a promising target for esophageal cancer treatment. Combining miR-452-3p inhibition with APC agonists may synergistically suppress esophageal cancer progression, a strategy worthy of preclinical investigation.

This study has several limitations. First, single-center recruitment with a small sample size may cause selection bias. Second, in vivo experiments are required to confirm the role of miR-452-3p in tumor growth and metastasis. Third, the downstream signaling pathways of the miR-452-3p/APC axis in esophageal cancer still require further investigation. We plan to conduct multi-clinical sample experiments and establish esophageal cancer animal models to further investigate the molecular mechanisms by which miR-452-3p regulates esophageal cancer. Concurrently, this will predict the interactions between the miR-452-3p/APC axis and other pathways.

In summary, miR-452-3p upregulation in esophageal cancer correlates with tumor progression and poor prognosis. By directly targeting APC to promote cancer cell malignancy, miR-452-3p may serve as a promising therapeutic target. However, its clinical applicability, safety, and regulatory mechanisms require further investigation.

## Supplementary Materials

Supplementary Material

## Figures and Tables

**Table 1.** Relationship Between the Expression of miR-452-3p in Esophageal Cancer Tissues and Clinicopathological Characteristics

**Variable**	**Cases (n = 155)**	**miR-452-3p Expression**		***P***
		**High (n = 79) **	**Low (n = 76) **	
Gender				.594
Male	87	46	41	
Female	68	33	35	
Age (years)				.216
<60	64	29	35	
≥60	91	50	41	
Histology				.473
Adenocarcinoma	31	14	17	
Squamous cell carcinoma	124	65	59	
Tumor diameter				.108
<3 cm	76	35	41	
≥3cm	79	44	35	
Differentiation				.196
Well + moderate	96	45	51	
Poor	59	34	25	
TNM stage				.019*
I-II	100	44	56	
III	55	35	20	
Lymph node metastasis				.026*
Yes	41	27	14	
No	114	52	62	

TNM, tumor–node–metastasis.

*P<.05.

**Table 2.** Cox Regression Analysis of Prognostic Factors in Patients with Esophageal Cancer

**Variable**	**HR**	**95% CI for HR**		***P***
		**Lower**	**Upper**	
miR-452-3p	2.653	1.189	5.916	.017*
Gender	0.622	0.273	1.413	.257
Age	1.394	0.666	2.916	.378
Histology	1.498	0.554	4.047	.426
Tumor diameter	2.026	0.713	5.755	.185
Differentiation	2.461	0.891	6.803	.082
TNM stage	2.938	1.105	7.812	.031*
Lymph node metastasis	2.392	1.048	5.460	.038*

TNM, tumor–node–metastasis. **HR, Hazard Ratio**

*P<.05.

## Data Availability

The data that support the findings of this study are available on request from the corresponding author.
