# Supplementary material for: miR-452-3p Targets Adenomatous Polyposis Coli to Regulate the Malignant Phenotypes and Prognosis of Esophageal Cancer
Source: Turk J Gastroenterol. 2026 May 13;37(7):749–56. doi: 10.5152/tjg.2026.26023 (PMC13382498; doi:10.5152/tjg.2026.26023)
Supplement: Supplementary Material [file supplementary_material.pdf]

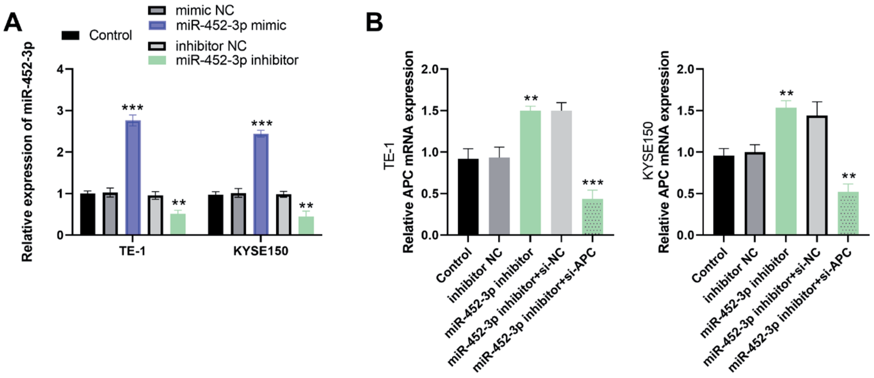

Supplementary Figure 1. Transfection results. (A) Changes in miR-452-3p levels following transfection. (B) Changes in APC levels following transfection. **$P < .01$, ***$P < .001$.

A

	Predicted consequential pairing of target region (top) and miRNA (bottom)
Position 3152-3159 of APC 3' UTR	5' ...AGAGAAGGUUGAUUUCAGAUGAA...
	|||||||
hsa-miR-452-3p	3' GUGAAUGAAGAAACGUCUACUC

B

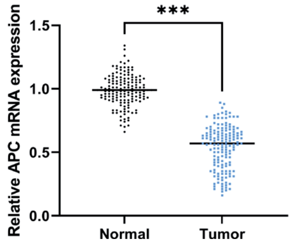

Supplementary Figure 2. (A) Predicted binding site of miR-452-3p in APC 3'UTR. (B) Expression of APC in esophageal cancer. ***$P < .001$.
